# The Preparation of Ag Nanoparticle and Ink Used for Inkjet Printing of Paper Based Conductive Patterns

**DOI:** 10.3390/ma10091004

**Published:** 2017-08-28

**Authors:** Lin Cao, Xiaohe Bai, Zhidan Lin, Peng Zhang, Shuling Deng, Xusheng Du, Wei Li

**Affiliations:** Institute of Advanced Wear & Corrosion Resistant and Functional Materials, Jinan University, Guangzhou 510632, China; linc19993@163.com (L.C.); bxh0625@163.com (X.B.); slingdeng@jnu.edu.cn (S.D.) xdusydjn@jnu.edu.cn (X.D.); liweijn@aliyun.com (W.L.)

**Keywords:** Ag nanoparticles, flexible electrode, Ag ink, inkjet printing

## Abstract

Ag nanoparticles were successfully prepared using a liquid reduction method with a suitable mixture reductant of polyethylene glycol (PEG) and ethylene glycol (EG). OP-10 as a dispersing agent, was used to prepare the conductive Ag ink. Ag nanoparticles with an average particle size of 40 nm were prepared while the ratio of PEG to EG was 1:2. Meanwhile, the Ag particles had a narrow size distribution and great dispersion performance. The effects of paper substrates, sintering temperature, and sintering time on the conductivity of the printed Ag ink pattern were also studied. It was found that Lucky porous high glossy photo paper was a good candidate as the printing substrate. The resistivity of the printed pattern could reach 5.1 × 10^−3^ Ω·cm after heated at 100 °C for 2 h. Hence, the printed pattern showed good conductivity which led to the LED light being on. Furthermore, the Ag nanoparticle ink could be printed to form any pattern as required that still showed good electrical conductivity after being sintered under low-temperature. This could provide new possibilities for the preparation of flexible electrodes.

## 1. Introduction

As a new type of functional material, Ag nanoparticles are widely used in many fields such as chemicals, aerospace, medical, electronics, and so on. With the development of large-area and flexibility of electronic products, Ag nanoparticles have broad application prospects, especially in the electronic and information manufacturing industries [1]. The liquid reduction method is the main method of preparing Ag nanoparticles (AgNPs) and presents great advantages including simple production equipment, convenient operation, and the low price of raw materials [2,3]. However, in the production process of silver powders, reaction parameters such as temperature, concentration of reducing agent, and amounts of capping agent are quite complex. As it is really difficult to control the size and shape of the silver powders, the value and application of Ag nanoparticles has been limited. Thus, the preparation of uniform, small sized, and single-shaped Ag nanoparticles rapidly and controllably is a current technology problem worldwide.

With the development of electronic printing technology, conductive thin films are widely used in flexible display circuit printing [4], solar photovoltaic cells [5], and light emitting diodes [6]. Paper substrate materials have great potential for use in portable electronic devices as they are flexible and portable. There are several methods for the deposition of conductive materials on a paper surface such as stamping, chemical vapor deposition, and spin coating [7]. However, to provide a smooth layer of conductive materials on solid substrates, these methods require a dust free environment and strictly controlled temperature, which are very expensive and difficult to maintain. As a form of digital printing, inkjet printing has developed quickly and has become more extensively applied. Compared to traditional printing methods, inkjet has a high utilization of materials, simple manufacturing process, low prices, large area, high precision, and many other advantages.

Generally, nano-Ag is the key functional material of the silver conductive ink and is composed of Ag particles, dispersants, surfactants, solvents, and other additives. Usually it is difficult to remove the dispersant and other additives at lower sintering temperatures because of their high boiling point. These additives severely decrease the conductivity of the printed pattern formed by the silver ink. To remove these additives, the sintering temperature of the conductive ink has to generally be higher than 200 °C [8], which severely restricts the use of certain flexible substrates such as paper [9], polyethylene terephthalate (PET) [10], and so on.

In our study, Ag nanoparticles were prepared using different mixing ratios of two reducing agents and various experimental parameters to study the reaction mechanism. Specific preparation schemes were proposed to obtain a good dispersal of Ag nanoparticles rapidly, which had different controlled particle sizes. A series of low boiling point additives were studied and introduced into the ink system for variety and to acquire a low sintered temperature conductive Ag ink for ink-jet printing.

## 2. Results

### 2.1. Characterization of AgNPs

**Figure 1:**
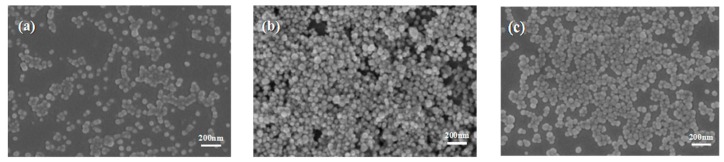
SEM images of Ag nanoparticles prepared under different temperatures: (**a**) 130 °C; (**b**) 140 °C; and (**c**) 150 °C.

**Figure 2:**
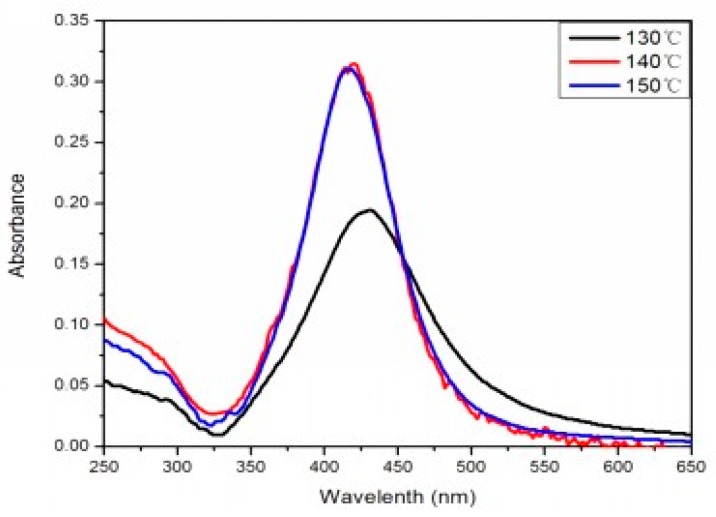
UV-vis extinction spectra of the products prepared at different temperatures at PEG:EG ratio of 1:2.

The morphology, particle size, and size distribution of Ag nanoparticles vary under different temperatures and reaction times so the effects of the volume ratios, temperatures, and time factors of the PEG-EG compound on reducing AgNPs were investigated. Figure 1a–c show the SEM image of AgNPs prepared under different temperatures. As shown in Figure 1, the prepared AgNPs were spherical. Figure 2 is the UV-vis extinction spectra of the products prepared under different temperatures when the volume ratio of PEG:EG was 1:2. When the reaction temperature was 130 °C, the UV-vis absorption band for AgNPs was around 440 nm. In addition, SEM photographs indicated that the average size was 100 nm. When the temperature was 140–150 °C, the UV-vis absorption band for AgNPs was around 420 nm. UV-vis diffuse reflection spectra results showed that absorb bands shifted to a shorter wavelength (blue shift), which was induced by the smaller particle size of AgNPs reduced in the solution. Figure 2 shows that the intensity of the peak at 420 nm for the samples aged at 140 and 150 °C was much stronger than that at 130 °C, mainly because a complete reaction is difficult to achieve under an insufficient temperature. Thus, it was difficult to obtain AgNPs with high yield, which was also verified by the SEM image. When the reaction temperature was 140 or 150 °C, the characteristic absorption peak had the same high and the half-peak had the same width on the plot graph. This indicated that 140 °C was a critical temperature of the reaction. When the temperature was higher than 140 °C, the rate of the reaction was almost constant. The morphology and particle size characterized by SEM remained essentially unchanged, and the photographs indicated that the average size of the AgNPs was 40 nm. Therefore, resources could be saved and quality assured under the selected temperature of 140 °C.

**Figure 3:**
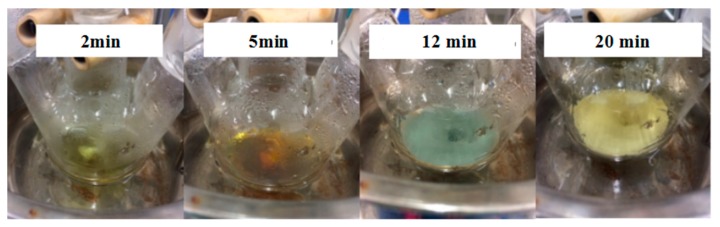
The color change during the preparation of Ag nanoparticles.

**Figure 4:**
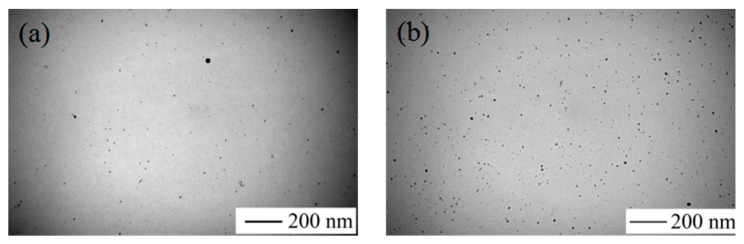

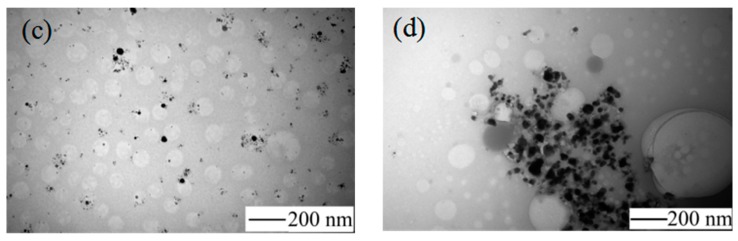
TEM images of the preparation of Ag nanoparticles. (**a**) 2 min; (**b**) 5 min; (**c**) 12 min; and (**d**) 20 min.

The results shown in Figure 3 demonstrate that the color of the solution changed obviously. The color of the solution gradually changed from colorless to yellow within 2 min. Figure 4 shows the TEM image of AgNPs and Figure 4a shows the appearance of the condensation nuclei. The solution mixture continued to be stirred after 3 min at the same temperature, and the color of the solution gradually changed to tea red before turning dark and cloudy, due to the increase in scattering degree of the crystal seeds after the growth of the light. After 12 min, the color became dark green, and the Ag nanoparticles grew from the condensation nucleus (Figure 4b–d). After 20 min, the color remained basically stable as brown green, which indicated that the reaction was complete as the absorption and scattering of light intensity are constant when the grain size is no longer growing.

**Figure 5:**
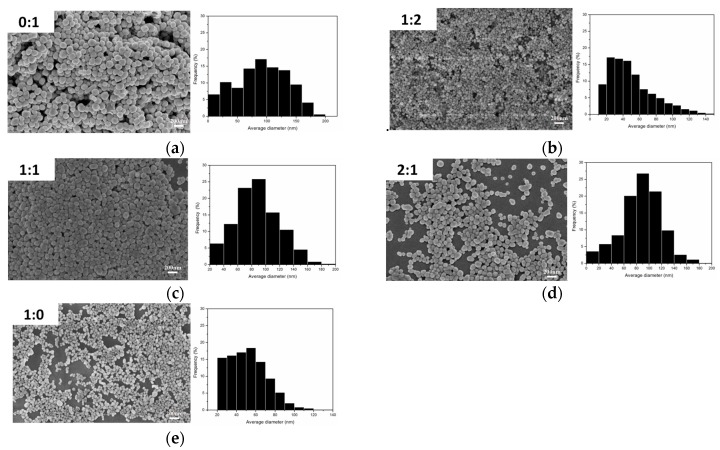
SEM images and particle size distribution images of Ag nanoparticles prepared under different volume ratios of PEG to EG. (**a**) PEG:EG = 0:1; (**b**) PEG:EG = 1:2; (**c**) PEG:EG = 1:1; (**d**) PEG:EG = 2:1; and (**e**) PEG:EG = 1:0.

Figure 5 show the SEM images and particle size distribution images of Ag nanoparticles prepared under different volume ratios of PEG to EG where it can be seen that the nano-Ag particles were well spread. Figure 5a–c show the SEM image of the AgNPs with an average size of about 65–100 nm with a wide size distribution. When the ration of PEG:EG was 1:2 (Figure 5b), the average nano-Ag size was about 40 nm, and the particle size distribution was concentrated within 30–80 nm, which was an ideal ratio in this series of experiments. Figure 5e shows the SEM image when the ratio of PEG:EG was 1:0; the corresponding size distribution result is displayed and show a mean diameter of 40 nm. However, this ratio also had some disadvantages, for example, PVP and AgNO_3_ were barely soluble in PEG, had a low yield, took a long time for reaction processes, and had incomplete reactions. The results showed that the ratio (1:2) of PEG to EG were more suitable. The addition of PEG effectively inhibited the growth rate of Ag particles and also effectively controlled the size of nano-Ag particles by adjusting the ratio of PEG to EG. PEG also prevented the agglomeration of Ag particles so that the prepared nano-Ag particles had good dispersibility.

**Figure 6:**
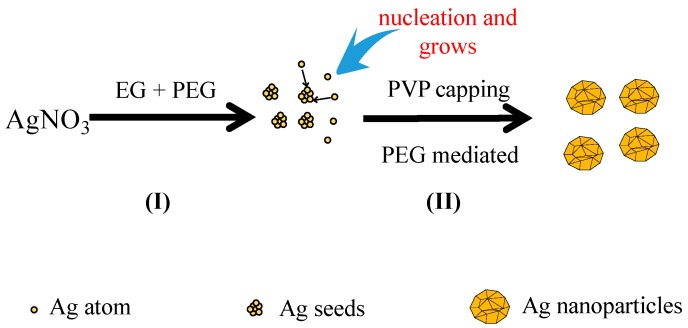
Schematic illustration of formation of uniformity Ag nanoparticles in mixed solvents of EG and PEG.

The growth mechanism of the spherical Ag nanoparticles in the mixed solution of PEG and EG was proposed as Figure 6I. When EG was used alone as the reductant, the nano-Ag formed very slowly due to the weak reducing power of EG. In addition, selective adsorption of PVP onto nano-Ag was improved, which resulted in the formation of various morphologies of nano-Ag in the reaction system such as nanowires and nanocubes. PEG was employed to tune the morphology of nano-Ag as PEG has a different reducing ability and viscosity from that of EG. On one hand, due to the stronger reducing power of PEG than EG, its addition would accelerate the reaction rate, and the concentration of Ag^+^ in the solution decrease quickly and reach to a supersaturated status. If the reduction rate was very fast, the newly formed Ag atoms could be quickly deposited onto the nuclei without affecting the formation of new nuclei. According to the conservation of mass, the atomic weight of the whole system was constant. As more Ag atoms were involved in nucleation, less of them would take part in the formation of new nuclei, and consequently the number of nuclei per unit time increased [11]. On the other hand, as shown in Figure 6II, the high viscosity of PEG reduced the movement speed of the grains in the solution and the possibility of collision among the grains decreased. Thus, the nucleation and growth rate of Ag increased with the increasing reduction ability of PEG, which facilitated the formation of numerous relatively uniform and large crystals. In contrast, if the reduction ability of the reductant was weak, the nucleation and growth rate were slow. Therefore, employing the reductant mixture of PEG and EG in the reaction led to fast nucleation and slow growth rates to obtain a single morphology of nano-sized particles.

**Figure 7:**
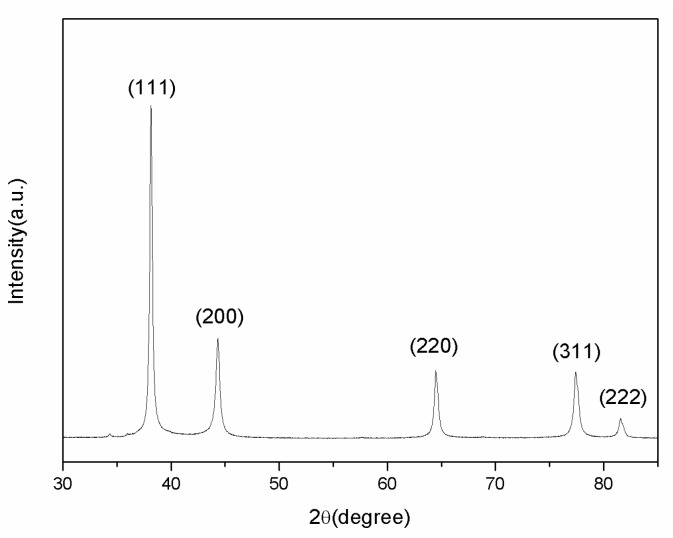
XRD pattern of Ag powder samples.

XRD analysis was carried out to verify the component of the prepared AgNPs. As shown in Figure 7, the significant diffraction peaks at 2-theta angle of 38.12°, 44.3°, 64.5°, and 77.5° were assigned to the (111), (200), (220), and (311) planes of face-centered-cubic (fcc) silver (JCPDS file No. 04-0783), respectively.

### 2.2. Characterization of Ag Conductive Patterns

**Figure 8:**
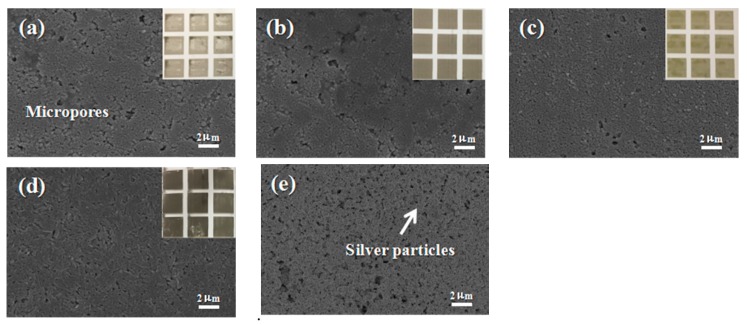
The SEM of the printed Ag patterns using different photo papers as substrates and the surface structure of different photo papers. (**a**) Epson; (**b**) Kodak; (**c**) Fantac; (**d**) Lucky; and (**e**) the Lucky photo paper printed Ag patterns.

The optimum conductivity of AgNPs on different photo paper substrates such as EPSON, Lucky, FANTAC, and Kodak was investigated. The fabrication of conductive patterns and patterned anodes are illustrated in Figure 8. A personal computer was used to design conductive patterns which were then inkjet-printed with a home printer (Canon iP1188, Canon, Tokyo Daejeon, Tokyo, Japan). Ink concentration is important for inkjet printing as a much higher concentration can block the nozzles, and much lower concentration may increase the printing times for acquiring appropriate electrodes. Therefore, 15 wt % Ag was chosen for the printed ink. Figure 8a–d show the photo images of different paper substrates. The optical images of the photo paper showed a homogenous and thin coating of AgNPs on the photo paper. To verify the homogeneity of the AgNPs thin coating on the photo paper, a series of surface morphology SEM images of different photo papers were taken. As shown in Figure 8a–d. The conductivity of AgNPs on the different paper substrates was tested by measuring their respective resistance, which all showed a high value of conductivity. The Lucky photo paper was chosen for the preparation of the electronic circuit and electrodes due to its flexibility, smooth surface, fine pore size, and moderate absorption of the ink. Other paper substrates were unevenly coated with ink stains as the large sized pores of these paper surfaces led the AgNPs to spread and become trapped among the paper fibers, causing a discontinuity of the conductive track. Therefore, the Lucky photo paper was selected for electrode preparation. Figure 8e shows the SEM of the Lucky photo paper printed Ag patterns, and the macroscopic picture is provided in Figure 8d.

**Figure 9:**
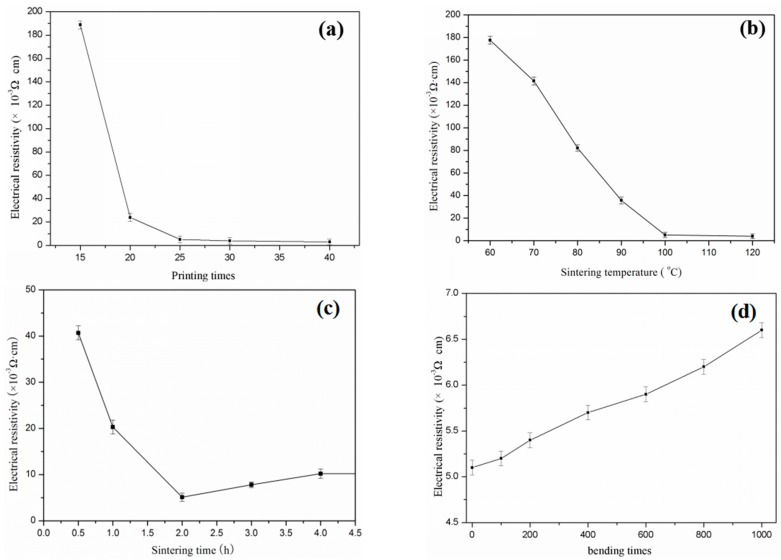
The resistivity of the printed Ag patterns with: (**a**) different printing times; (**b**) different sintering temperature; (**c**) different sintering time; and (**d**) different bending times.

The Ag ink was used to inkjet conductive patterns. The electrical resistance reduced rapidly with the increasing number of overprinting. It was also observed that adding Ag nanoparticles significantly improved conductivity, which was mainly due to the good conductivity of Ag nanoparticles and the good connection between the Ag and photo paper. After being printed 40 times (Figure 9a), the conductive pattern fabricated by the Ag ink formed a 4 × 10^−3^ Ω·cm sheet resistivity. However, the decreasing resistivity was not obvious after being printed 25 times, making 25 the best number of printed times.

Figure 9b,c show the relationship between the resistivity of the printed Ag patterns and the sintering temperature, resistivity, and sintering time, respectively. The variation of sintering temperature and time are the most important parameters that affect the resistivity of AgNPs on the substrate materials. The stability and conductivity of the electronic track was tested by changing the sintering temperatures from 60 to 120 °C. The pattern was printed 25 times and sintered for 120 min. The best conductivity that the thin film AgNPs obtained was when the sintering temperature was 120 °C, as sintering temperatures over this decreased resistivity. The sintering time also impacted on the conductivity of the AgNPs pattern on the photo paper substrate. The effects of sintering time on conductivity were studied by heating the printed track at 120 °C with different interval times of 0.5, 1, 2, 4, and 6 h. The results of the conductivity of AgNPs at different sintering time are given in Figure 9c. A high conductivity was acquired when the sintering time was 2 h; moreover, there was no damage to the paper substrate after heating. It was best to obtain a maximum conductivity of AgNPs on the paper substrate. After the sample was bended 1000 times, conductivity decreased by 29.4% (Figure 9d). 

**Figure 10:**
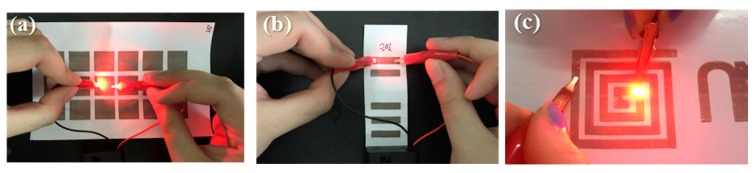
The simple conductivity test of printed Ag patterns: (**a**) Square; (**b**) Rectangle; (**c**) Spiral.

Furthermore, Ag conductive patterns were used to demonstrate the lighting of an LED bulb using a 9 V battery, as shown in Figure 10. This result demonstrated that the prepared conductive track could be used as an electronic circuit and also applied in other electronic applications.

## 3. Materials and Methods 

### 3.1. Materials

All chemicals used in this study were of analytical reagent grade. Meanwhile, Ag nitrate, polyvinyl pyrrolidone (PVP), ethylene glycol (EG), polyethylene glycol (PEG), and isopropanol (IPA) were obtained from the Tianjin Damao Chemical Reagent Factory (Tianjin, China). Ethyl alcohol was provided by the Guangzhou Chemical Reagent Factory (Guangzhou, China). Octylphenol polyoxyethylene ether (10) (OP-10) was provided by the Tianjin Fuyu fine chemical industry (Tianjin, China).

### 3.2. Synthesis of AgNPs

PEG and EG were mixed for use as a reducing agent. The preparation steps of spherical nano-Ag particles were as follows. First, 100 mg of AgNO_3_ and 150 mg PVP was transferred into a round-bottomed flask containing 20 mL of EG. Next, 20 mL of PEG was added to the stirred mixture, and combined until well mixed. The mixed solution was then transferred to an oil bath with a constant temperature and 200 r/min constant stirring. At the completion of the reaction, the mixture was washed and diluted with anhydrous ethanol 5 times before concentrated products were achieved after 20 min centrifugation at 8000 rpm. The liquid had obvious stratification, with the lower layer having a higher AgNPs concentration. The precipitated product was cleaned and centrifuged with anhydrous ethanol 5–8 times. The precipitated product was dried at room temperature standby.

### 3.3. Synthesis of Nano Ag Ink

To synthesize the new Ag nano-ink, the recipe used was as follows: 3 wt % of OP-10; 20 wt % of ethylene glycol; 1.5 wt % ethanol; and 1 wt. % isopropyl alcohol was placed into glass bottles containing 59.5 wt % deionized water. The solution mixture was stirred at 200 r/min constant speed. After 10 min, 200 mg of AgNPs was added to the mixed solution which was then ultrasonically cleaned for 30 min, and the mixture formed a uniform system at room temperature when it was stirred for 30 min. Finally, a uniform AgNPs ink was obtained. The AgNPs ink was injected into a clean ink cartridge (Canon iP1188, Canon, Tokyo Daejeon, Tokyo, Japan) and printed using a Canon iP1188 inkjet printer. To deposit a large number of AgNPs, the printer settings were adjusted for the “best print quality” and “photo print mode” to ensure sufficient AgNPs ink was printed on the paper substrate.

### 3.4. Characterization of AgNPs

Morphological observations were carried on using a field-emission scanning electron microscope (FE-SEM, Zeiss, Oberkochen, Germany) and a field-emission transmission electron microscope (TEM, Philips TECNAI-F20, Chur, Switzerland). Samples were also analyzed by X-ray diffraction (XRD; D8, Bruker, Billerica, MA, USA) at room temperature with a scanning angle from 20° to 90°. The scanning speed was 10°/min with 40 kV accelerating voltages and a 15 mA current. A UV-Vis absorption spectroscopy test was conducted using a UV-Vis spectrophotometer (UV-2550, Japan Shimadzu Corporation, Kyoto, Japan). The sample was diluted 1000 times and placed in a 1 × 3 cm quartz cuvette for testing. The resistance of the thin AgNPs pattern was tested using a KDB-1 digital four-probe resistance tester (Guangzhou Kunde Technology Companies (Guangzhou, China)).

## 4. Conclusions

In summary, based on a controlled reaction temperature, ratio of PEG to EG, and reaction time, the Ag nanoparticles were prepared through a liquid reduction method. PEG and EG were mixed as reducing agents to prepare silver nanoparticles with a range of different particle sizes under 140 °C. When the ratio of PEG to EG was 1:2, silver nanoparticles were prepared with an average particle size of 40 nm, a narrow size distribution and good dispersion performance. The conductive AgNPs ink was produced using OP-10 as a dispersing agent. The influence of other parameters was also studied, such as the effects of paper substrates, sintering temperature and time on the conductivity of the printed pattern. It was found that the Lucky porous high glossy photo paper was a good candidate substrate, and the resistivity of the printed pattern after heating at 100 °C for 2 h could reach 5.1 × 10^−3^ Ω·cm. When the printed pattern was connected to an LED light and supplied the power, the LED lit up. Therefore, it was possible to print any patterns as designed, had good electrical conductivity, and could be sintered at a low-temperature and has provided new ideas for the preparation of flexible electrodes.
